# An exciton-polariton bolometer for terahertz radiation detection

**DOI:** 10.1038/s41598-018-28197-0

**Published:** 2018-07-04

**Authors:** G. G. Paschos, T. C. H. Liew, Z. Hatzopoulos, A. V. Kavokin, P. G. Savvidis, G. Deligeorgis

**Affiliations:** 1 Department of Materials Science & Technology, University of Crete, 71003 Heraklion, Greece; 2 FORTH IESL, Heraklion, 71110 Crete, Greece; 3 Division of Physics and Applied Physics, School of Physical and Mathematical Sciences, Nanyang Technological University, 637371 Singapore, Singapore; 4 School of Physics and Astronomy, University of Southampton, Highfield, Southampton, SO17 1BJ United Kingdom; 5 Spin Optics Laboratory, St. Petersburg State University, 198504 Peterhof, St. Petersburg Russian Federation; 6 CNR-SPIN, Viale del Politecnico 1, I-00133 Rome, Italy; 7 ITMO University, 197101 St. Petersburg, Russian Federation

## Abstract

We experimentally investigate the feasibility of a bolometric device based on exciton-polaritons. Initial measurements presented in this work show that heating – via thermal expansion and bandgap renormalization – modifies the exciton-polariton propagation wavevector making exciton-polaritons propagation remarkably sensitive to thermal variations. By theoretical simulations we predict that using a single layer graphene absorbing layer, a THz bolometric sensor can be realized by a simple exciton-polariton ring interferometer device. The predicted sensitivity is comparable to presently existing THz bolometric devices with the convenience of being a device that inherently produces an optical signal output.

## Introduction

Bolometers are well-known for their ability to detect electromagnetic radiation by absorbing energy and measuring associated temperature changes^1^. Typically, the read-out translates the signal to electrical resistance due to thermal effects in the sensing material or to polarization in pyroelectric materials. Bolometer arrays dominate the market for infrared imaging due to their low cost and ease of manufacturing, nevertheless, bolometer systems are still an active research area aiming at improved terahertz (THz) detection capability^2^. In particular, micro- bolometers with metallic films deposited on thin membranes have resulted in commercial THz cameras^3,4^. The main drawback is their limited sensitivity compared to more expensive cryogenically cooled systems. As applications of THz technologies^5^ are becoming more common place, the development of future THz detectors, such as antenna-coupled bolometers^6^ and graphene-based devices^7^ is gaining ever increasing interest.

In a seemingly separate area of research, semiconductor microcavities with quantum wells create a fusion of electronic and optical properties in the form of exciton-polaritons. While traditionally studied for their fundamental effects^8^, these quasi-particles have demonstrated a number of properties useful for hybrid electro- optic devices: direct coupling to both electric and optical fields, fast-response times, long coherence lengths/times, and strong nonlinearity as compared to typical nonlinear optical materials. Polariton based switches^9,10^, transistors^11–13^, and electro-optic control^14,15^ have been reported in recent years. A variety of theoretical studies have also identified polaritons as prominent generators of THz radiation, by making use of the bosonic final state stimulation of THz frequency transitions^16–21^ or oscillations between multiple quantum wells^22,23^.

Although in the recent years, numerous polariton based THz emitting devices have been proposed, until now, no polariton based THz detection schemes have been considered. In contrast to conventional bolometer schemes, a polaritonic based bolometer sensor would couple THz induced thermal variations to a polariton-based signal rather than an electrical output. The long coherency of polaritons would then enable an additive effect over the device size leading to increased sensitivity. A few related studies are available for inspiration: initially, thermally- induced switching of polaritons was reported^24^, where polariton response was found in reasonable timescales of a few hundred picoseconds when relying on heat propagation over distances of a few tens of microns. Additionally, the coherence of polaritons was best illustrated by the experimental realization^25^ of a polariton ring interferometer^26^ with size on the order of a few tens of microns. Polaritons were readily created either by directly exciting the system by a resonant laser or formed spontaneously through polariton Bose-Einstein condensation^27,28^ or lasing^29,30^, realizing a highly phase sensitive system. Furthermore, cooling and heating actions of polariton lasers have been recently experimentally demonstrated^31,32^. Finally, it was recently proposed that semiconductor microcavities could be coupled to graphene^33^, which is a good THz frequency absorber due to its gapless energy spectrum.

Based on the above constituents, we realize an exciton-polariton based interferometric device utilizing one-dimensional channels to propagate coherent polariton condensates. We experimentally measure the temperature induced changes in the interference of counter-propagating polariton condensates and show that such a bolometer device can serve as a platform for temperature sensing and consequently be adopted for use as a THz radiation detector.

Based on experimental findings of polaritons’ transport in such model system, we theoretically analyze a ring interferometer device and estimate the detection sensitivity of such a polariton based Terahertz bolometer. For this we consider the scheme in which a polariton condensate ring interferometer is formed by etching a planar semiconductor microcavity structure^12,25^ to realistically examine the feasibility of the proposed polariton ring bolometer. Remarkably, the estimated sensitivity is at par with the current state of the art electronic bolometers with the added advantage of being - to the best of our knowledge - the first design to output a coherent photonic signal.

## Results

### Temperature sensitivity of interfering exciton-polaritons

**Figure 1:**
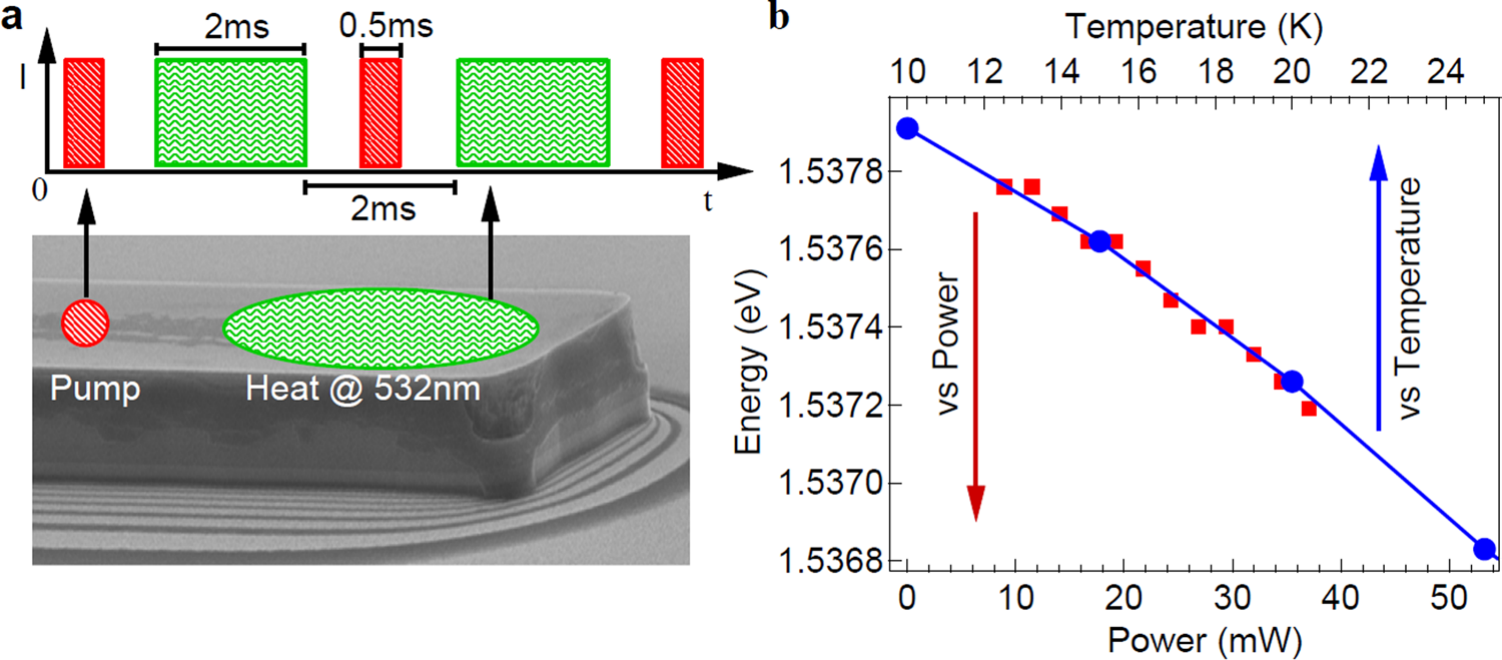
Pump and heating laser spots together with their arrival times on the ridge. Ridge temperature calibration. (**a**) Ridge formed by plasma etching of a planar microcavity structure. Polariton condensate created by the pump laser (red spot) propagates towards the end of the ridge. A second heating laser (532 *nm*) is used near the edge (green spot) to raise the temperature of the ridge, the spot size is comparable to the ridge size to ensure uniform heating. The duration of the excitation and heating lasers are, respectively. The heating laser is turned off while interference measurements are taken to avoid any effect due to spurious carriers creation. (**b**) For establishing direct relation between heating laser power and ridge temperature, lower polariton energy shifts are recorded by changing the heat laser power (bottom axis) and by varying cryostat temperature (top axis).

To experimentally assess the temperature effect on the exciton-polariton condensate coherent transport, a 20 *μm* wide and 300 *μm* long ridge was formed via reactive ion etching of a high-finesse planar microcavity^12^. An electron micrograph of the ridge is shown in Fig. 1a. The ridge waveguide was placed in a closed cycle-cryostat and excited by two separate laser beams. A 5.3 *mW* non-resonant pulsed pump laser centered at 1.649 *eV*, with a repetition rate of 87 *MHz* and a 2 *μm* diameter spot size was focused at a distance of 40 *μm* away from the edge to generate the condensate flow. A second Nd:Vanadate (Nd:YVO_4_) CW laser at 532 *nm* with a 20 *μm* diameter spot, was used to locally induce temperature changes near the ridge edge. Initially, the correspondence between heating laser power and induced temperature change was evaluated by recording lower polariton emission energy shifts as a function of varying heating laser power. The corresponding emission shift is shown in Fig. 1b, in red data points. Subsequently, the cryostat temperature was slowly varied and the corresponding Photoluminescence (PL) emission energy was recorded in the absence of heating leaser. The blue dots and the top x axis in Fig. 1b correspond to the recorded energy and the associated temperature respectively. The described procedure enabled precise calibration of temperature changes (top x-axis) induced by the heating laser power (bottom x-axis).

**Figure 2:**
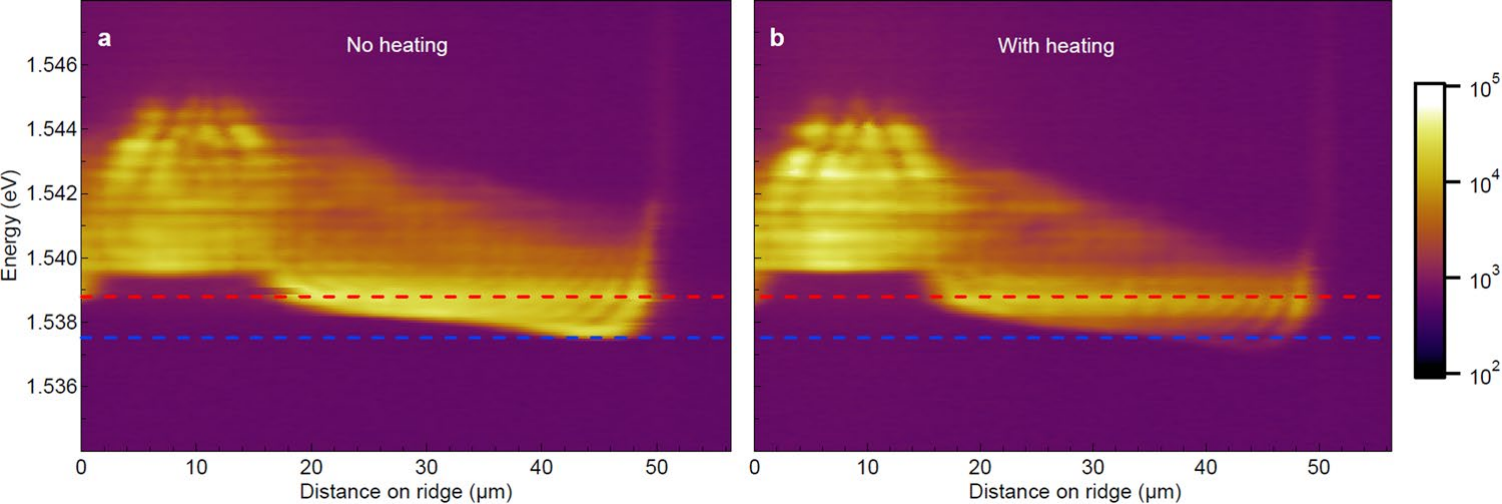
Polariton emission energy vs ridge position cross-section is shown with heating laser off and on. (**a**) No heating. (**b**) With heating. Interference pattern due to back-reflected polaritons can be seen in both cases showing that the heating does not affect coherency of the propagating polaritons.

**Figure 3:**
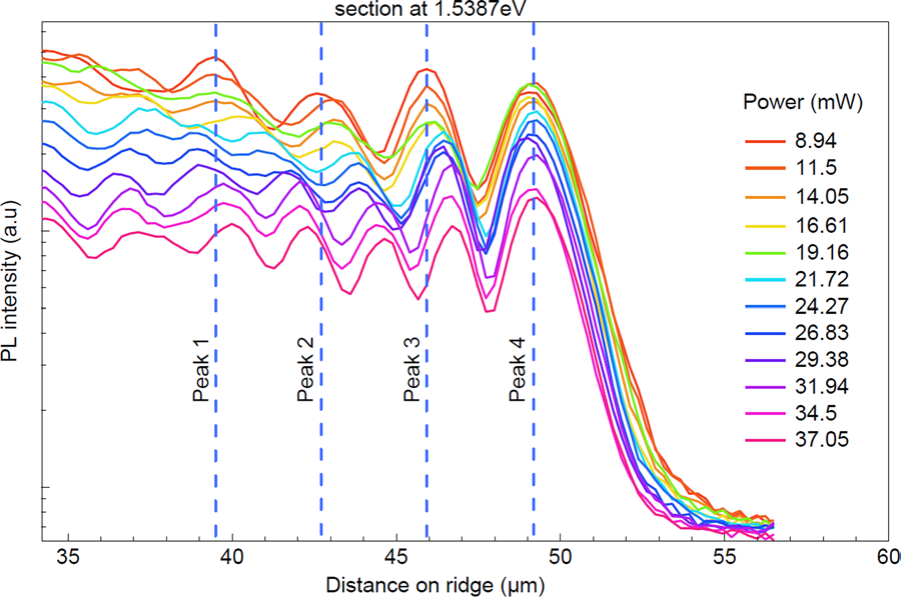
PL intensity vs distance on the ridge at 1.5387 *eV* for increasing heating power. PL intensity at fixed energy that corresponds to the red dashed line in Fig. 2 at 1.5387 *eV* for varying heating power. The beating due to interference of forward and backward propagating polariton condensates can be seen. Changes in the beating frequency are observed with the increasing heat laser power.

The pump laser produces polaritons that propagate through the structure and reflect at the edge of the ridge. The set of counter propagating coherent polaritons form an interference pattern as shown in Fig. 2. That presents an energy resolved real space emission across the ridge. By varying the heating laser power, control of the local temperature near the edge of the ridge is achieved. The interference pattern persists even at the highest heating laser powers of 37.05 *mW*, as can be seen from the two images recorded without (Fig. 2a) and with (Fig. 2b) heating. To ensure the heat laser does not create any spurious carriers affecting the condensate flow during measurement, the two beams are temporally separated as shown in the top schematic of Fig. 1a. To achieve this, the heating laser was modulated using a mechanical chopper with 4 *ms* period and on time of ~2 *ms* (50% duty cycle). Inside the time window when the heating was switched off, the pump laser was turned on for ~0.5 *ms* generating the condensate flow. Assuming that the heat laser generated carrier lifetime is less than a nanosecond, it is safe to assume that polaritons will only respond to temperature changes induced by the heating laser. In Fig. 2, clear changes in the polariton interference pattern due to heating can be observed. Such drifting interference patterns can be clearly identified in Fig. 3, where fixed energy cross-sections at 1.5387 *eV* (shown by the red dashed line in Fig. 2) are extracted from the emission taken as a function of heating power.

**Figure 4:**
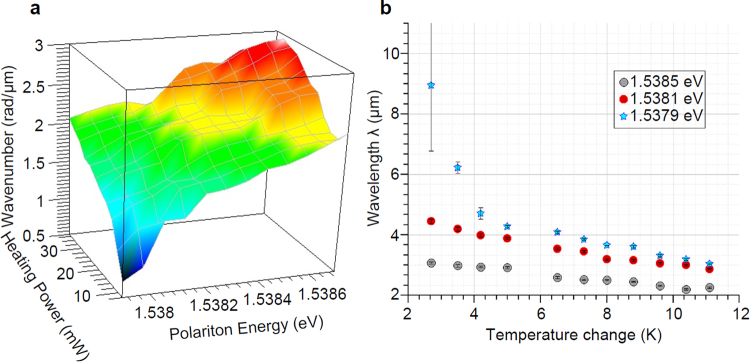
3D plot of special frequency and polariton beating wavelength changes vs temperature. (**a**) A three dimensional plot of spatial frequency as a function of polariton energy and heating power. The highest change is observed for low power and low energy. (**b**) A plot of polariton beating wavelength for selected polariton energy as a function of the temperature change due to heating.

It is evident that changing the temperature induces a change in polariton propagation wavevector and thus a change in beating frequency of the resulting interference. The beating frequency is a function of the temperature - i.e. the heating power - and the energy at which the PL was measured. This spatial frequency was extracted for each case by fitting the standing wave oscillations with a sin(*kx*) where *k* is expressed in *rad/μm* and plotted in Fig. 4a as a function of both heating power and polariton energy. In Fig. 4b, heating power is correlated to temperature variation and *k* = *2 π/λ* is solved for *λ*, so the extracted *λ* in *μm* was plotted as a function of temperature. From the latter figure, it is shown that the rate of change *dλ/dT* depends on the selected polariton energy as well as the heating power ranging from *dλ/dT* = *3.4* *μm/K* for polariton energy of 1.5379 *eV* to *dλ/dT* = *0.04* *μm/K* for polariton energy 1.5385 *eV*. The previously discussed results were obtained for a polariton source – edge distance of 40 *μm*. Further experiments have verified that interference is observable at source – edge distances as high as 140 *μm* without loss of contrast, suggesting that the energy resolved detection scheme used in our experiments is not suffering from loss of coherence due to scattering even at large distances.

### Theoretical modeling of a polariton ring detector

**Figure 5:**
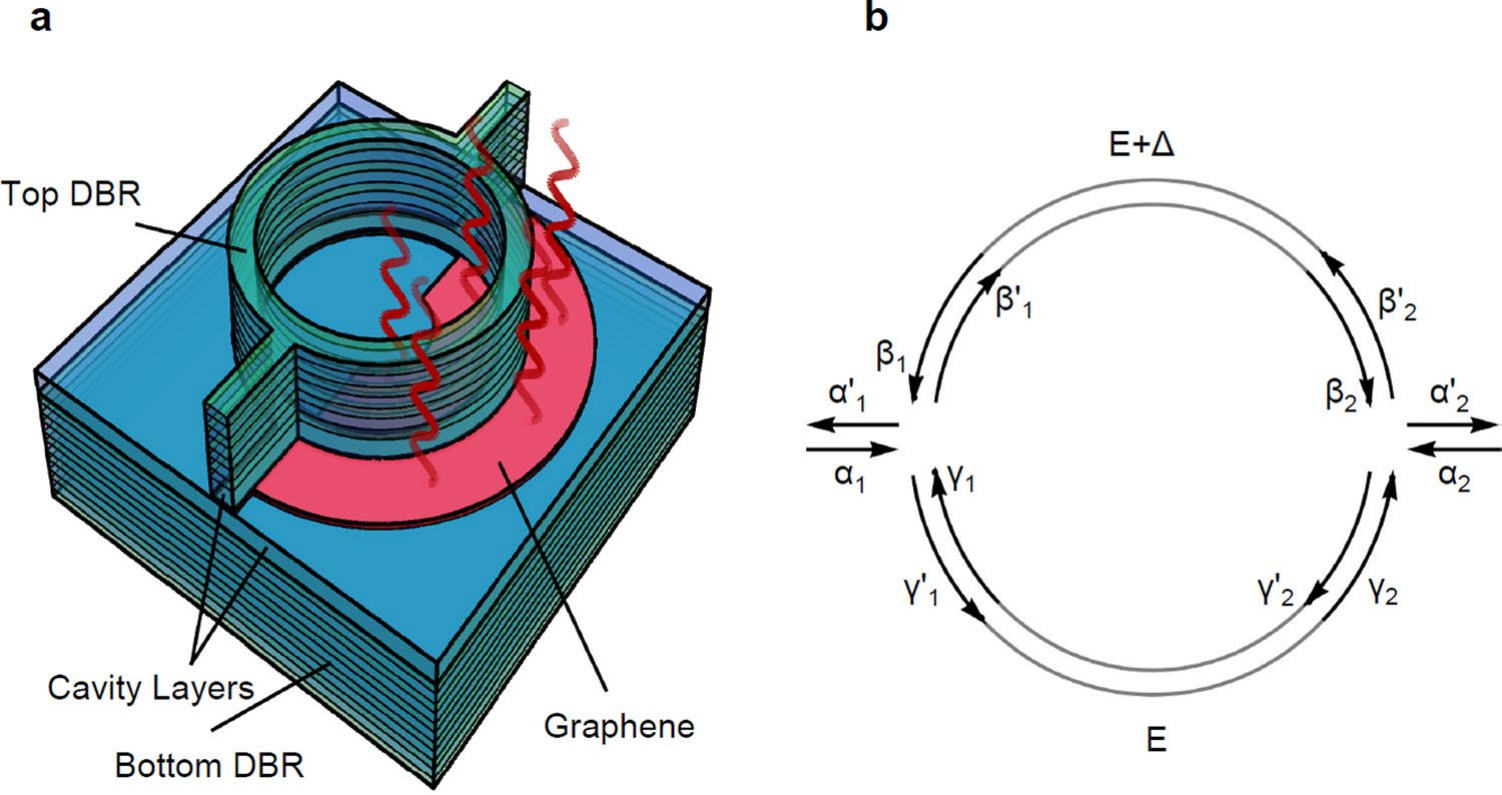
Polariton bolometer scheme and propagating field amplitudes in the polariton ring interferometer. (**a**) Schematic of a polariton bolometer, composed of a polariton ring interferometer with one arm heated by a THz absorbing graphene layer. (**b**) Forward and backward propagating field amplitudes in the polariton ring interferometer.

We now consider theoretical estimates of the sensitivity of a polariton ring-interferometer, illustrated in Fig. 5. In this geometry, polaritons injected at one port (left) of the ring would split and take different paths around the ring. Their interference at the exit channel would determine their transmitted intensity. Any phase difference accumulated between the two arms would strongly affect the phase of the recombining polariton stream at the exit port (right) and thus result in varying transmission. Placing a layer of graphene around one arm of the interferometer would allow it to absorb any incident terahertz radiation and thus produce a temperature shift. We know that this will introduce a change in the polariton wavelength, in accordance with the experimental measurements of the previous section. To determine the sensitivity of the polariton transmission to the incident terahertz field, we need to: (1) determine how small a temperature change can be detected; (2) determine how much terahertz radiation must be absorbed to induce the required temperature change; and (3) Determine how much incident terahertz radiation is needed, accounting for inevitable reflections and losses.

#### Ring interferometer temperature sensitivity

To determine how strong a temperature change an exciton-polariton ring bolometer would require, we can model the interference of polaritons propagating through a polariton ring using a transfer matrix formalism^34^. We begin by identifying three input fields and three output fields at each of the junctions of the ring, as shown in Fig. 5b. The input fields can be injected resonantly^9–11^ or non-resonantly^12^ making use of polariton condensation^25^. In either case the result is that polaritons are coherent, and have a definite phase.

The relationship between the fields at each of the junctions is determined by a scattering matrix^34^:

1 $$(\begin{array}{c}{\alpha ^{\prime} }_{1,2}\\ {\beta ^{\prime} }_{1,2}\\ {\gamma ^{\prime} }_{1,2}\end{array})=S(\begin{array}{c}{\alpha }_{1,2}\\ {\beta }_{1,2}\\ {\gamma }_{1,2}\end{array}),$$

where due to symmetry we assume that the scattering matrix is the same for the left and right junctions. The scattering matrix can be written as:

2 $$S=(\begin{array}{ccc}-(a+b) & {\sigma }^{1/2} & {\sigma }^{1/2}\\ {\sigma }^{1/2} & {a} & b\\ {\sigma }^{1/2} & b & a\end{array}),$$

where *a* and *b* are respectively the amplitude and reflection coefficients in the ring at the junctions. *σ* is the coupling coefficient to input and output channels, related to a and b by flux conservation^34^, and can be expressed as $a=\pm \,\frac{1}{2}(\sqrt{1-2\sigma }-1)$ and $b=\pm \,\frac{1}{2}(\sqrt{1-2\sigma }+1)$. The matrix S thus depends on a single parameter σ, which would be given by the specific geometry of the etched nanostructure at the junctions.

The left and right junction fields are related by:

3 $$(\begin{array}{c}{\beta }_{1}\\ {\beta }_{2}\end{array})=(\begin{array}{cc}{e}^{ikr\pi } & 0\\ 0 & {e}^{ikr\pi }\end{array})(\begin{array}{c}{\beta ^{\prime} }_{2}\\ {\beta ^{\prime} }_{1}\end{array}),$$

4 $$(\begin{array}{c}{\gamma }_{1}\\ {\gamma }_{2}\end{array})=(\begin{array}{cc}{e}^{ik^{\prime} r\pi } & 0\\ 0 & {e}^{ik^{\prime} r\pi }\end{array})(\begin{array}{c}{\gamma ^{\prime} }_{2}\\ {\gamma ^{\prime} }_{1}\end{array}),$$

Polaritons pick up a phase upon traversing each of the interferometer arms, which we assume to be semicircular with radius r. The phase depends on the tangential component of the polariton wavevector. We take the wavevector *k* = *2 π/λ* for polaritons in the upper arm of the ring and *k*′ = *2 π/(λ* + *δλ)* for polaritons in the lower arm of the ring, where λ and *λ* + *δλ* are the wavelengths of polaritons in the upper and lower arms of the ring, respectively. We now have relations between all quantities depicted in Fig. 5b. By setting α_2_ = 0 we can thus calculate the polariton intensity transmission coefficient, ${|{\alpha ^{\prime} }_{2}/{\alpha }_{1}|}^{2}$ with the definitions $\phi =k\pi r$ and $\phi ^{\prime} =k^{\prime} \pi r$:

5 $${|\frac{{\alpha ^{\prime} }_{2}}{{\alpha }_{1}}|}^{2}=\frac{4{\sigma }^{2}{(sin\phi +sin\phi ^{\prime} )}^{2}}{({\sigma }_{+}{({\sigma }_{-}cos(\phi -\phi \text{'})+2(\sigma -1)cos(\phi -\phi \text{'}))}^{2}+4{\sigma }^{2}si{n}^{2}(\phi +\phi \text{'}))}$$

where we defined:

6 $${\sigma }_{+}=1-\sigma +\sqrt{1-2\sigma }$$

7 $${\sigma }_{-}=1-\sigma -\sqrt{1-2\sigma }$$

**Figure 6:**
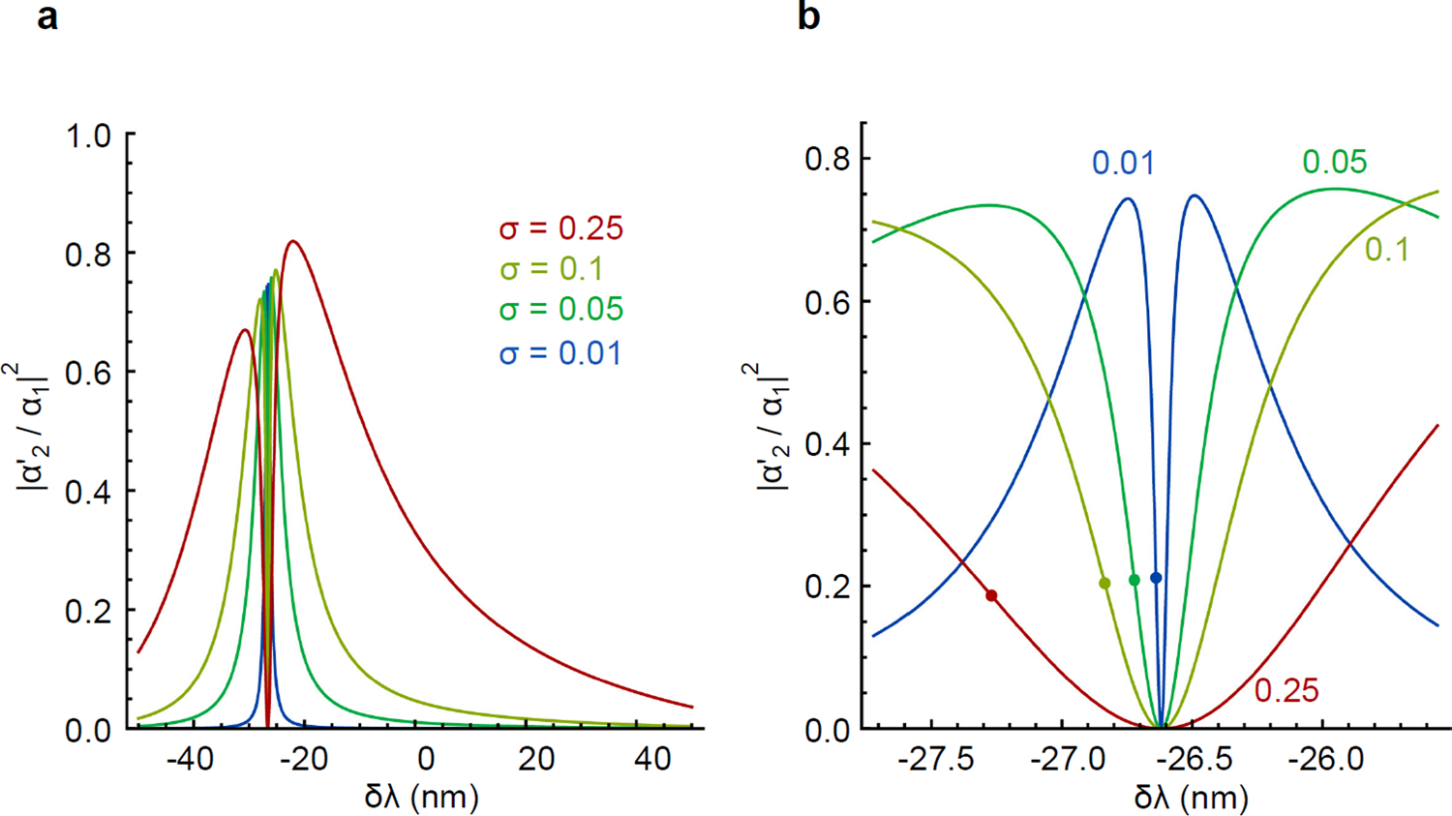
Polariton transmission as a function of wavelength shift. (**a**) Polariton transmission through a polariton ring interferometer as a function of the upper arm wavelength shift, *δλ*. (**b**) Close-up of the center of the resonance peak in (**a**). Points correspond to the strongest transmission gradients. Parameters: r = 50 *μm*, λ_0_ = 5 *μm*.

The transmission is shown in Fig. 6 as a function of the wavelength shift *δλ* assuming a ring radius of 50 *μm*. Observable transmission changes are available over a wide range of coupling parameters σ. The gradient of transmission is strongest for non-zero δλ, which corresponds to a point of destructive interference (including multiple loop contributions). To enhance the sensitivity it makes sense to operate the system at destructive interference by shifting the wavelength – or equivalently the size – of the lower arm by a constant amount δ_0_, to the optimum points marked in Fig. 6b. Remarkably, very small wavelength changes, down to tenths of nm are capable of a significant change in transmission. Using an experimentally determined value for *dλ/dT*, we obtain temperature change sensitivity of 10^−5^ *K* in a ring of 50 *μm* radius.

#### THz absorbed power needed for a temperature gradient

To describe heat propagation in the considered sample plane we can consider the heat equation:

8 $$D{\nabla }^{2}u(x,y)=\frac{-q(x,y)}{{c^{\prime} }_{p}\rho ^{\prime} {d}_{z}},$$

For simplicity we assume a two-dimensional (2D) geometry, where any heat that escapes the quantum well plane is assumed to be dissipated. On the left-hand side of Eq. 8, *u(x, y)* is the temperature distribution and *D* is the thermal diffusivity of GaAs, which is given by $D=\frac{K}{{c}_{p}\rho }$, where *K* = 1400 *W·m*^*−1*^*K*^*−1*^ is the thermal conductivity, C_p_ = 2.7 *J·Kg*^*−1*^*K*^*−1*^ is the specific heat capacity, and *ρ* = 5316.5 *kg·m*^*−3*^ is the material density.

The right-hand side of Eq. 8 depends on the heating rate $q(x,y)\approx f(x,y){P}_{||}$, where $f(x,y)$ is an envelope function describing the shape of the THz absorbing layer and *P*_*||*_ is the power absorbed per unit area. ${c^{\prime} }_{p}$, $\rho ^{\prime}$ and ${d}_{z}$ correspond to the specific heat capacity, material density and thickness of the THz absorbing material, respectively. For single-layer graphene^35^ ${c^{\prime} }_{p}=10\,Jk{g}^{-1}{K}^{-1}$, $\rho ^{\prime} =2,000\,kg{m}^{-1}$, and ${d}_{z}\approx 0.34\,nm$.

The Green function that satisfies Eq. 8 is:

9 $$G(x,x^{\prime} )=-\,1/4\pi Dvx-x^{\prime} v,$$

Finally, the temperature field is then given by:

10 $$u(x,y)=\frac{-{P}_{||}}{{c^{\prime} }_{p}\rho ^{\prime} {d}_{z}}\int G(x,x^{\prime} )f(x^{\prime} )dx^{\prime} ,$$

**Figure 7:**
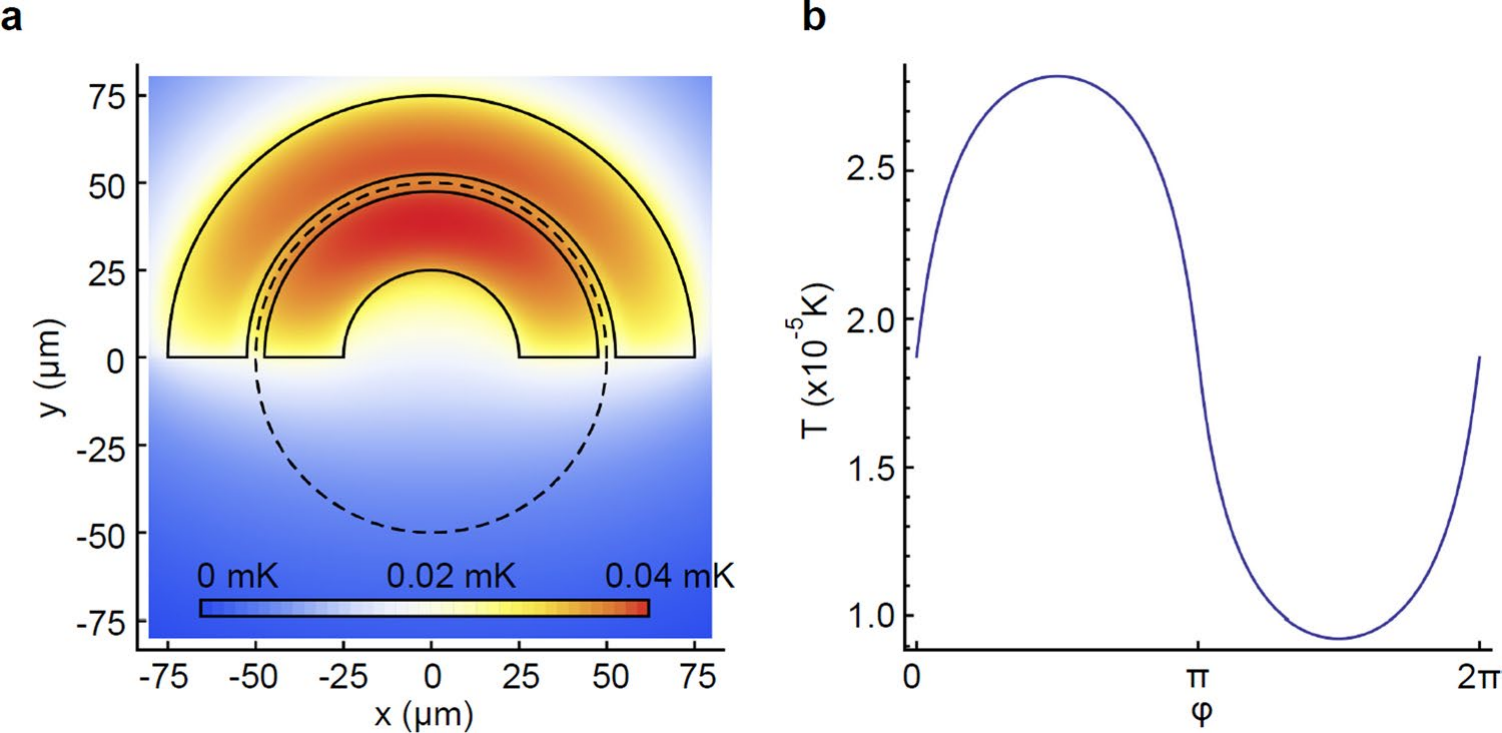
2D temperature map of two heated semicircular annuli and temperature difference as a function of the radial position on the ring. (**a**) 2D temperature map of two semicircular annuli (marked by solid black boundaries), heated with a power of P_||_ = 0.1 *mW·cm*^*−2*^. (**b**) Temperature difference around the ring corresponding to the dashed circle in (**a**).

Considering a system of two semi-circular annuli, for an absorbed power of *P*_||_ = 0.1 *mW·cm*^*−2*^ we obtain the temperature variation shown in Fig. 7a.

Figure 7b shows that the considered power is sufficient to induce a temperature difference of 10^−5^ K between the two semi-circular annuli of the considered system.

Required incident THz power: The dielectric function of graphene in the terahertz range can be represented with the Drude model:

11 $$\varepsilon (\omega )=1-\frac{{\omega }_{p}^{2}}{\omega (\omega -i\gamma )},$$

where, *ω*_*p*_ and *γ* are the plasma frequency and damping rate, respectively. Various values of these parameters have been reported in the literature^36^. Here we take a set of parameters corresponding to a very conservative estimate of the THz absorption by graphene^37^: *γ* = 1 *meV and ω*_*p*_ = *1.2* *eV*. We note that the absorption could be significantly higher^36^, particularly for chemically or electrically doped samples^38^.

Given the dielectric function, the real refractive index is $n(\omega )=\sqrt{(\varepsilon +R\{\varepsilon \}/2)}$, which corresponds to an intensity reflection coefficient, $R(\omega )={|(n(\omega )-1)/(n(\omega )+1)|}^{2}$. The imaginary component of the dielectric function gives the THz absorption coefficient as, $\alpha (\omega )=2\omega I\{\varepsilon (\omega )\}/c$, with *c* the speed of light. Finally, we expect the absorbed energy fraction to be:

12 $$\frac{{P}_{{\rm{abs}}}}{{P}_{{\rm{inc}}}}=(1-R)(1-{e}^{-a{d}_{z}})$$

For a THz photon energy ${\hbar }\omega =1\,meV$, we find P_||_/P_inc_ = 1%, suggesting that an impinging THz power of *P*_inc_ = 0.1 *mW·mm*^*−2*^ would induce a sufficient temperature shift to achieve a measurable response.

In principle, the bolometer could also absorb in the radio frequency range. However, significant radio absorption by graphene typically requires special engineering of the structure^39^ and typical radio wave intensities are less than the considered range of terahertz intensities. Also, in principle, radio wave frequencies could be filtered out to avoid unwanted interference of a THz detector.

#### Combined analysis and comparison to other devices

**Figure 8:**
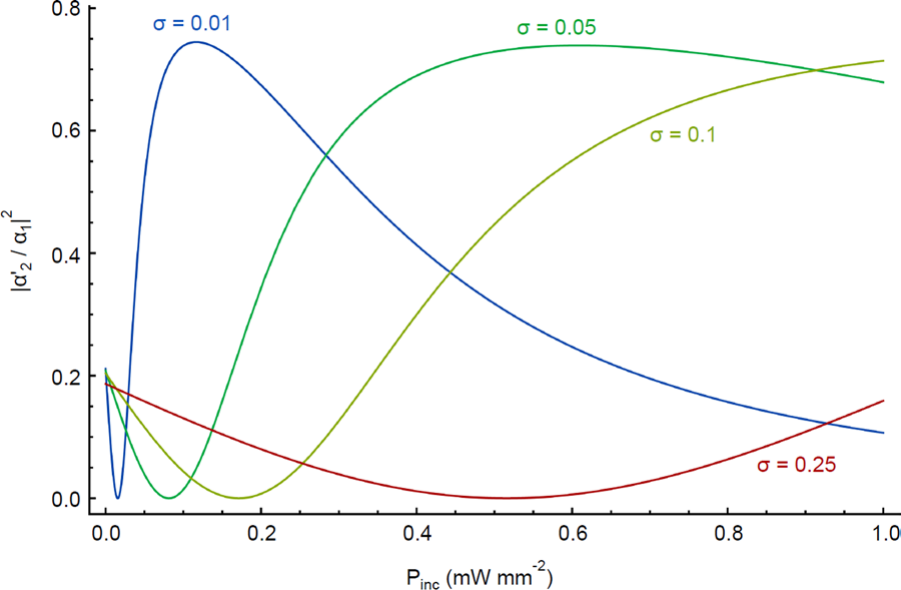
Polariton transmission as a function of incident terahertz power. Dependence of transmission on incident terahertz power, taking into account an angle-dependent wavelength from power-dependent temperature maps derived from Fig. 7. We assumed that the wavelength changes linearly with temperature, with *dλ/dT* = 3.4 *μm/K*, in agreement with our experimental results (section 2).

The combined result of our analysis is shown in Fig. 8, which predicts that terahertz intensities of around *P*_inc_ = 0.1 *mW·mm*^*−2*^ are detectable via a change in polariton transmission. For our device absorption area $(\pi \times {75}^{2})/2\,\mu {m}^{2}$, this corresponds to sensitivity of less than 10^−6^ *Watt* of terahertz power.

For comparison, electronic bolometer terahertz detectors have been developed for some time and they are typically compared based on their noise equivalent power. Comparison of different devices^40^ have shown that optimum devices have a noise equivalent power of $3\times {10}^{-10}W/H{z}^{1/2}$. To put this into context, a device operating at ~10 *MHz* signal frequency would be capable of detecting of ~10^−6^ W THz power. The considered exciton-polariton bolometer response speed is limited mainly by the arm heating response that given the small size of the device is estimated to be in the order of 10 MHz. We thus conclude that the polariton based bolometer can match the sensitivity of electronic systems (for a given repetition rate).

The main advantage of our device however is not necessarily to provide a high terahertz absorption sensitivity, but rather to demonstrate a terahertz induced modulation of a polariton signal compatible with previous demonstrations of exciton- polariton based information processing. The use of graphene as a terahertz absorber maintains possibilities for further development of tunable devices^41^ and, if necessary, higher detection sensitivity could be achieved by using bilayer or multilayer graphene^42,43^. Also, the possibility of polariton flat-bands may allow further enhanced response to temperature-induced energy shifts^44–46^.

## Discussion

Polaritonic devices exploit the unique quantum and optical properties of exciton-polaritons, superposition quasiparticles combining large coherence lengths (comparable with the coherence length of a semiconductor laser) and strong dependence on the parameters of the crystal lattice that affect their excitonic component. We considered the detection of terahertz frequency radiation using a graphene-exciton-polariton based bolometer, which takes advantage of both features of exciton-polaritons: it would rely on the interference in a flow of polaritons propagating through a ring and a possibility to suppress this interference by shifting the exciton resonance frequency, which we found experimentally to be highly sensitive to the lattice temperature in a one-dimensional channel geometry. These experimental results support a theoretical analysis predicting the feasibility of graphene-exciton-polariton based bolometers.

## Methods

### Sample preparation

The sample used in the present work is a high quality factor semiconductor microcavity. The microcavity structure consists of a bottom and a top *5λ/2* AlAs/AlGaAs distributed Bragg reflector (DBR) of 35 and 32 layer pairs respectively and are formed by molecular beam epitaxy (MBE), while the active medium is composed by four sets of three 10 *nm* AlGaAs/GaAs quantum wells placed at the antinodes of the cavity electric field. Microcavity ridges of 20 *μm* wide and 300 *μm* long are formed via reaction ion etching on the high-Q microcavity, providing lateral confinement for the propagating polaritons along the surface of the ridges. Etching of the top DBR is followed by deposition of a thin gold layer over the etched area, thus reducing unwanted background emission from the non-etched active region quantum wells surrounding the ridge.

### Data availability

The datasets generated during and/or analysed during the current study are available from the corresponding author on reasonable request.
